# Dialkylated dibenzo[*a*,*h*]anthracenes for solution-processable organic thin-film transistors†

**DOI:** 10.1039/d4ra07439d

**Published:** 2024-11-14

**Authors:** Tengzhou Yang, Liang Zhang, Yucong Bao, Haoming Wei

**Affiliations:** a School of Physics and Physical Engineering, Qufu Normal University Qufu Shandong 273100 China yangtz@qfnu.edu.cn

## Abstract

Here, two alkylated dibenzo[*a*,*h*]anthracene (DBA) derivatives with linear *n*-dodecyl (C12-DBA-C12) and ring-containing pentyl-cyclohexyl (Cy5-DBA-Cy5) moieties were successfully synthesized. Their chemical and thermal stability were both notably high, with the molecular arrangement of C12-DBA-C12 and Cy5-DBA-Cy5 being greatly influenced by the alkyl groups. C12-DBA-C12 formed a 2D lamellar herringbone packing structure and its blade-coated film exhibited high layered crystallinity and high carrier mobility up to 2.97 cm^2^ V^−1^ s^−1^. By contrast, the arrangement of Cy5-DBA-Cy5 in the crystal exhibited a packing motif where π-cores and alkyl chains were intertwined due to the C–H⋯π proximity of cyclohexyl moieties and DBA cores. Meanwhile, Cy5-DBA-Cy5 demonstrated relatively poor film-forming capacity and moderate mobility of about 0.45 cm^2^ V^−1^ s^−1^. These findings could expand the possibilities of using DBA instead of pentacene in developing high-performance OSCs for organic electronics, and offer insights into manipulating molecular arrangement through alkyl engineering.

## Abstract 2

Here, two alkylated dibenzo[*a*,*h*]anthracene (DBA) derivatives with linear *n*-dodecyl (C12-DBA-C12) and ring-containing pentyl-cyclohexyl (Cy5-DBA-Cy5) moieties were successfully synthesized.

## Graphical abstract

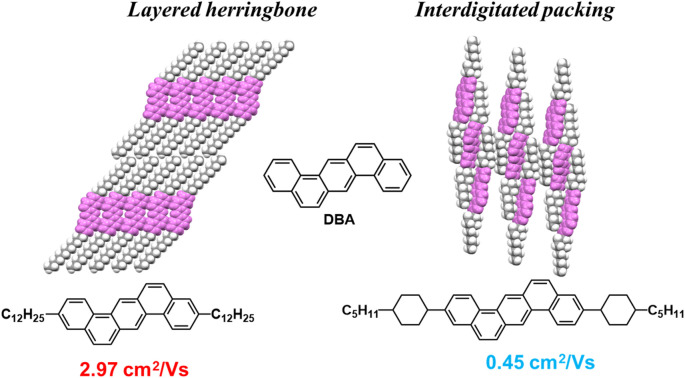

## Introduction

Fused polycyclic aromatics,^1–10^ having extended π-conjugated structures, are highly significant for applications in organic thin-film transistors (OTFTs) because they facilitate the carrier transport by enhancing the intermolecular interactions between adjacent molecules. Among them, pentacene, a benchmark molecule with five linearly-fused benzene rings, has been extensively investigated in p-channel OTFTs,^11–17^ demonstrating high hole mobility exceeding 10 cm^2^ V^−1^ s^−1^.^18,19^ However, the relatively high HOMO level (−5.0 eV) of pentacene leads to its chemical instability and degradation in air,^20,21^ which severely deteriorates its OTFT performance. Furthermore, poor stability also limits the chemical modification process to develop new pentacene derivatives that have sufficient solubility and semiconducting functionality. Changing the fusion configuration of benzene rings is an effective technique to lower the chemical reactivity of aromatic systems.^22–24^ Following the above method, picene, in which benzene rings are fused in a W-shaped fashion, has been extensively studied in OTFTs. Indeed, picene and its derivatives were found to have the expected stability at ambient condition, coupled with a relatively high mobility exceeding 1 cm^2^ V^−1^ s^−1^.^25–27^ But, picene's transistor characteristics are highly sensitive to atmospheric conditions, particularly in the presence of oxygen.^25^

Recently, dibenzo[*a*,*h*]anthracene (DBA)^28,29^ is being highlighted as an option for electronic systems that are comparably stable, featuring a unique N-shaped molecular structure with five benzene rings angularly fused. In terms of OTFTs, DBA has not received much attention. A recent investigation^29^ demonstrates that DBA offers several benefits for OTFTs compared to its isomeric counterparts, pentacene and picene. On one hand, with a deep HOMO level of −5.7 eV, DBA had higher ambient stability than pentacene. On the other hand, OTFTs made with asymmetric DBA derivatives didn't exhibit the operational sensitivity in air, which is usually observed in picene OTFTs. Furthermore, a remarkable hole mobility of 11.4 cm^2^ V^−1^ s^−1^ was discovered in a Ph-DBA-C8 device revealing the good carrier-transport ability of DBA core.^29^ However, poor solubility (less than 0.5 mg mL^−1^ in chlorobenzene even at 60 °C) of these asymmetric DBA compounds hindered their accessibility of large-area, uniform crystalline films at low temperature.^29^ In light of DBA's high ambient stability and favorable carrier-transport characteristics, it is worthwhile to develop new DBA-based OSCs that have high mobility and sufficient solvent solubility. Among various chemical modifications, it is widely accepted that long-alkyl-chain substitution on π-core is an effective method for not only increasing solubility but also fine-tuning the intermolecular π–π packing. Moreover, recently, cyclohexyl-containing alkyl groups demonstrated their good use in DNTT, resulting in high thermal stability and exceptional OTFTs mobility of 15 cm^2^ V^−1^ s^−1^.^30,31^ This is probably because of the enhanced layered structure and film-forming ability by the introduction of cyclohexyl groups. But, a systematical study on how cyclohexyl-type alkyls influence the molecular arrangement has not been involved. Such cyclohexyl-type substitution may be a useful design strategy for achieving high-performance OSCs.

**Fig. 1:**
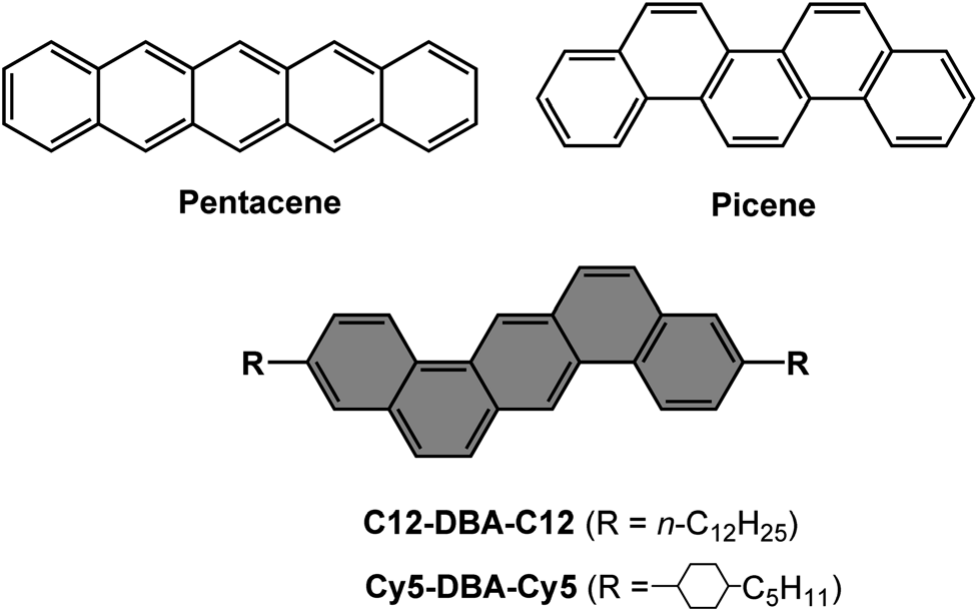
Chemical structures of pentacene, picene and R-DBA-R derivatives in this study.

Therefore, in this study, we reported two kinds of DBA derivatives, named as R-DBA-R (R = C12, Cy5), in which linear (*n*-C_12_H_25_, C12) and ring-containing (pentyl-cyclohexyl, Cy5) alkyl chains were symmetrically franked into DBA core at the end positions (Fig. 1). The effects of two different alkyl moieties on their solubilities, thermal properties, molecular assemblies, OTFTs characteristics and thin-film crystallinities were systematically studied. They both exhibited good chemical and thermal stability. In addition, C12-DBA-C12 possessed greater ability to dissolve in aromatic solvents compared to Cy5-DBA-Cy5. We found that C12-DBA-C12 formed the typical layered herringbone packing structure showing highly-ordered crystallinity and high mobility, whereas Cy5-DBA-Cy5 exhibited an antiparallel π-core-alkyl-interdigitated packing motif with relatively poor film crystallinity and low mobility. These findings suggest that DBA could serve as an alternative for pentacene, and our study can offer guidance for developing high-performance OSCs by alkyl engineering.

## Results and discussion

### Synthesis

**Scheme 1:**
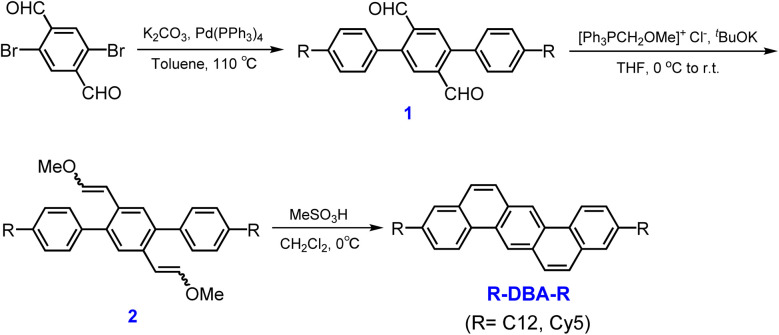
Synthetic route of C12-DBA-C12 and Cy5-DBA-Cy5.

Scheme 1 displayed a simple approach for synthesizing two dialkylated DBA derivatives with *n*-dodecyl and cyclohexyl-pentyl substituents. Starting with 2,5-dibromoterephthalaldehyde, the dialdehyde intermediates 1 were produced by a palladium-catalyzed Suzuki coupling reaction with the corresponding alkylphenylboronic acids. Then, using ^*t*^BuOK as a base, it can be readily transformed into the bis-methoxyviny isomers 2 by the Wittig reaction with chloro(methoxymethyl)triphenylphosphine. Finally, under the presence of an excess of MeSO_3_H, the intramolecular cyclization of 2 proceeded smoothly yielding the target compounds C12-DBA-C12 and Cy5-DBA-Cy5. Both of the final products were isolated by column chromatography and purified by recrystallization to obtain high-purity crystalline samples for subsequent measurements and device evaluations.

### Solubility and thermal stability

Solvent solubility is a key parameter for solution-processable OSCs. The solubilities of the C12-DBA-C12 and Cy5-DBA-Cy5 were evaluated in anisole and chlorobenzene. As displayed in Table S1,† they were more soluble in chlorobenzene than in anisole, and C12-DBA-C12 substituted with linear *n*-dodecyl chains had better solubility than Cy5-DBA-Cy5 with ring-containing pentyl-cyclohexyl ones. At an elevated temperature (60 °C), the solubility of C12-DBA-C12 in chlorobenzene rapidly increased up to 15.38 mg mL^−1^, approximately four times higher than that of Cy5-DBA-Cy5 (4.44 mg mL^−1^). Compared to the previously reported asymmetric DBA OSCs, superior solubility was successfully realized in the dialkylated C12-DBA-C12 and Cy5-DBA-Cy5 in this study. Additionally, the thermal stabilities of C12-DBA-C12 and Cy5-DBA-Cy5 were examined by thermogravimetric analysis (TGA). Both of them exhibited extremely high stabilities with the 5% loss temperatures (*T*_d_) over 395 °C (Fig. S1†). Furthermore, it was found that *T*_d_ increased from 397 (for C12-DBA-C12) to 434 °C (for Cy5-DBA-Cy5), suggesting that the cyclohexyl group is more powerful in improving the intrinsic thermal stability than linear alkyl one.

### Optoelectronic and electrochemical properties

**Fig. 2:**
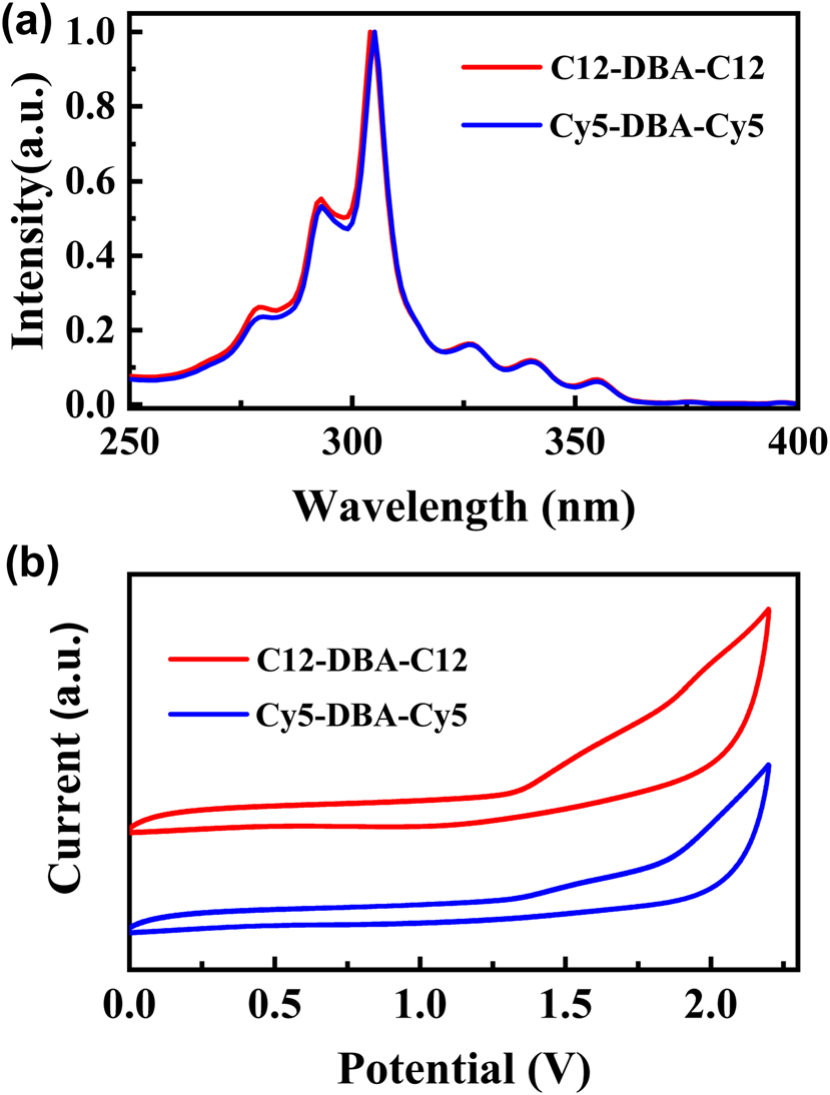
UV-vis absorption spectra (a) and CV curves (b) of C12-DBA-C12 and Cy5-DBA-Cy5.

As illustrated in Fig. 2a, the UV-vis spectra of C12-DBA-C12 and Cy5-DBA-Cy5 in dilute CHCl_3_ solution revealed the characteristic absorption bands of fused aromatics, such as *α*, *β*, and *para*-bands. As expected, their absorption was nearly identical to that of the DBA core with the absorption maxima located at 304 nm. Additionally, the optical band gaps (*E*_g_) of C12-DBA-C12 and Cy5-DBA-Cy5 estimated from the absorption edges were about 3.2 eV. Next, the cyclic voltammetry (CV) measurement was used to examine the electrochemical properties of two dialkylated DBA products. In Fig. 2b, they both showed similar irreversible oxidative waves, and their HOMO levels were estimated to be approximately −5.7 eV from the onset of the oxidation peaks, which is deeper than those of pentacene (−5.0 eV) and picene (−5.5 eV). These results indicated that C12-DBA-C12 and Cy5-DBA-Cy5 were chemically stable under ambient conditions.

### Single-crystal analysis

**Fig. 3:**
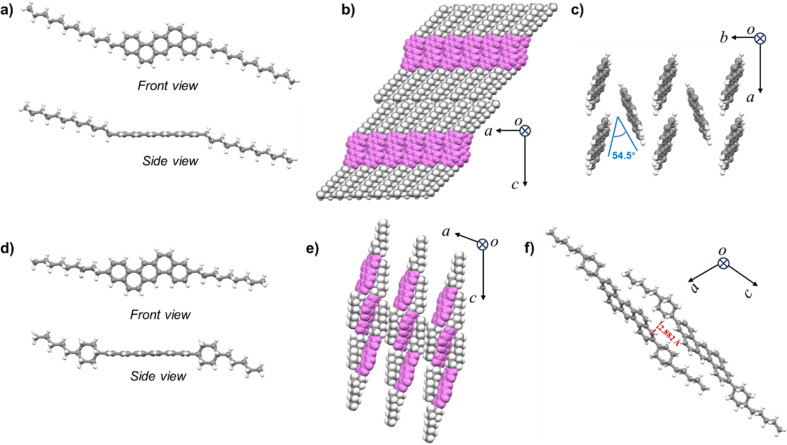
Molecular structures of (a) C12-DBA-C12 and (d) Cy5-DBA-Cy5. Packing structures of (b) C12-DBA-C12 and (e) Cy5-DBA-Cy5 (the DBA cores were marked in violet for clarity). (c) Herringbone motif of C12-DBA-C12 (the *n*-dodecyl chains are omitted for clarity) and (f) Cy5-DBA-Cy5 with short contacts.

In order to gain insight into the effects of alkyl conformations on the molecular and packing structures of C12-DBA-C12 and Cy5-DBA-Cy5, single-crystal structural analyses were performed. By the slow solvent evaporation from toluene, large plate-like single crystals of C12-DBA-C12 and Cy5-DBA-Cy5 were successfully obtained. As shown in Fig. 3a and d, the π-cores of two DBA compounds had identical planar geometries, while the alkyl chains that are either linear or cyclohexyl-containing stretched out with a typical antiperiplanar conformation. In addition, the cyclohexyl ring for Cy5-DBA-Cy5 was nearly perpendicular to the DBA core. The molecular arrangement differed significantly between C12-DBA-C12 and Cy5-DBA-Cy5, related to the different alkyl structures. As shown in Fig. 3b, C12-DBA-C12 crystallized in the triclinic *P*1̄ space group with a typical layered herringbone packing motif, which is preferable for 2D charge-carrier transport and occurs frequently in some high-mobility OSCs.^2^ The herringbone angle between the adjacent π-cores was 54.56° (Fig. 3c), close to that of pentacene (∼52°). By contrast, Cy5-DBA-Cy5 was found to crystallize in the monoclinic *C*2/*c* space group, featuring a unique interdigitated packing motif where the π-cores and the pentyl-cyclohexyl moieties interdigitated in opposite directions (Fig. 3e). It was apparent that there was almost complete displacement between adjacent π-cores, indicating the absence of carrier transport along the crystallographic *a*-axis. However, as displayed in Fig. S2.† another packing arrangement involving 1D slipping π–π interactions was found in the *b*–*c* plane of Cy5-DBA-Cy5 crystal, offering a pathway for carrier transport along the crystallographic *b*-axis. The proximity of cyclohexyl groups and DBA cores, which formed C–H⋯π contacts at a distance of 2.882 Å (Fig. 3f), inhibited the DBA cores from participating in π–π stacking, leading to the formation of this distinctive π-core-alkyl-interdigitated structure. The completely displaced π–π stacking observed in Cy5-DBA-Cy5 is quite different from that in the DNTT-based counterpart, in which adjacent DNTT cores arrange in a partially slipped packing ensuring effective π-orbital overlaps. It should be attributed to DNTT's larger π-conjugated system resulting in stronger π–π interactions. Therefore, in contrast to DNTT-based counterpart exhibiting ultrahigh mobility of 15 cm^2^ V^−1^ s^−1^, the completely-slipped packing of DBA cores in Cy5-DBA-Cy5 lead to its poor film-forming ability and thus nonideal carrier-transport properties, which were demonstrated in OTFTs section. Moreover, this intermolecular interaction led to a rise in the cohesive crystalline energy, which in turn caused Cy5-DBA-Cy5 to have relatively low solvent solubility. These findings indicated that linear alkyl groups are preferred for strengthening and maintaining the formation of layered herringbone crystalline structures.

### OTFTs characteristics

**Fig. 4:**
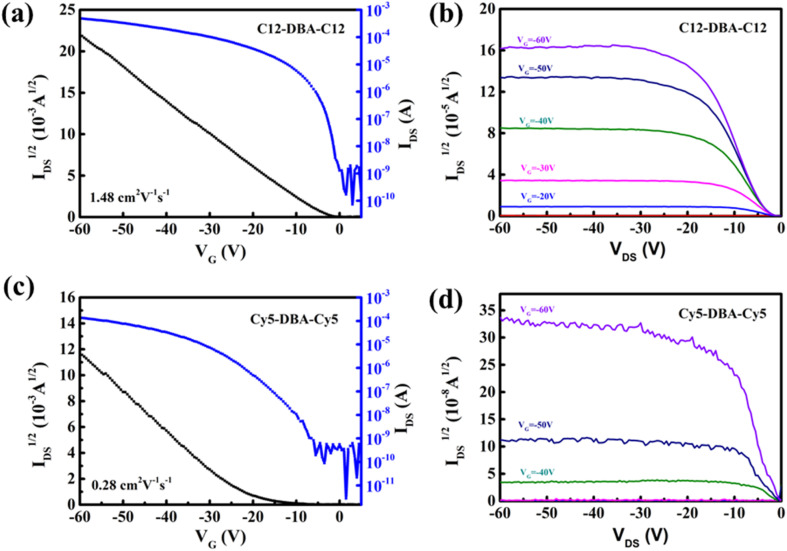
Transfer and output curves of (a and b) C12-DBA-C12, (c and d) Cy5-DBA-Cy5 on the BCB/SiO_2_ substrates.

**Fig. 5:**
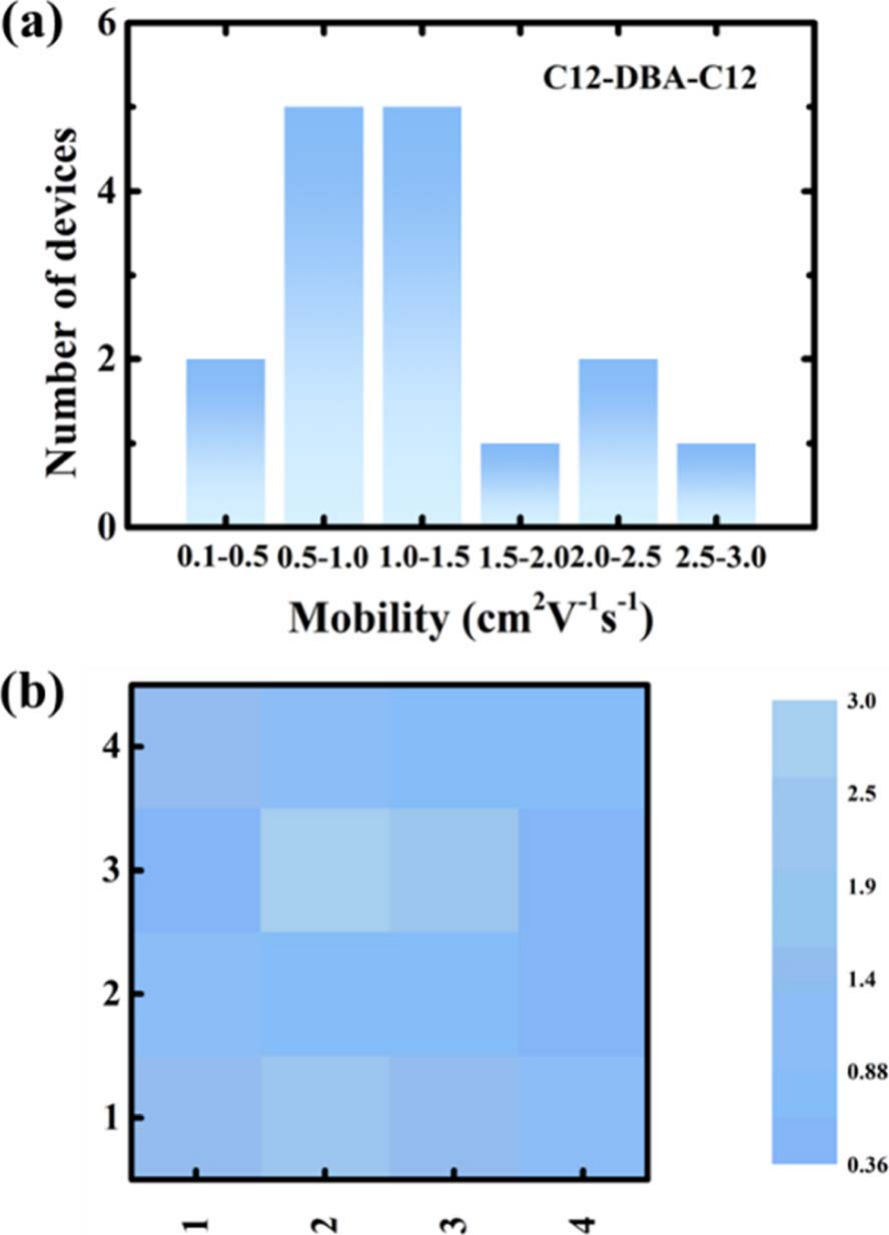
Distribution (a) and color map (b) of mobility of 4 × 4 device array on one BCB/SiO_2_ substrate.

To evaluate the carrier-transport capabilities of two DBA derivatives, OTFTs with bottom-gate/top-contact (BGTC) geometry were fabricated. The SiO_2_ gate dielectric layer was passivated by thermally cross-linked divinyltetramethyldisiloxane bis(benzocyclobutene) (BCB) polymer, and the total capacitance of BCB/SiO_2_ was estimated to be 9.1 nm cm^−2^ at low frequency (100 Hz, Fig. S3†). The crystalline films of C12-DBA-C12 and Cy5-DBA-Cy5 were deposited using a blade-coating method in anisole solutions. Their representative operating transfer/output curves and summarized OTFTs parameters were displayed in Fig. 4 and in Table 1, respectively. Obviously, the carrier-transport properties revealed the strong dependence on their molecular structures. As expected, C12-DBA-C12 OTFTs demonstrated satisfactory p-channel characteristics, including a maximum mobility of 2.97 cm^2^ V^−1^ s^−1^, on/off current ratios over 10^6^, as well as good drain-current saturation in the output curves. This is due to the high-quality crystalline films with favorable 2D herringbone packing structure. Conversely, the electrical properties of Cy5-DBA-Cy5 OTFTs were found to be relatively poor, with low mobilities (0.45 cm^2^ V^−1^ s^−1^) and on/off ratios (10^5^), and output curves with up-and-down noises. These characteristics are believed to stem from the absence of π–π stacking among neighboring Cy5-DBA-Cy5 molecules in the longitudinal molecular direction, and the poor film-forming ability probably caused by its low solubility. Furthermore, we fabricated and evaluated 16 OTFTs of C12-DBA-C12 on one BCB/SiO_2_ substrate (about 1 × 1 cm^2^ blade-coated film). As shown in Fig. 5, these transistors displayed consistent device-to-device uniformity with an average mobility of 1.22 cm^2^ V^−1^ s^−1^, underscoring the good film-forming ability of C12-DBA-C12. Overall, the newly-synthesized C12-DBA-C12 demonstrated impressive film-forming capacity by a solution process, achieving OTFTs mobilities that exceeded 1 cm^2^ V^−1^ s^−1^, comparable to those of the extensively studied pentacene and TIPS-pentacene.^2^ In addition, C12-DBA-C12 didn't exhibit the operational sensitivity in air, which is usually observed in picene OTFTs.^25^ Therefore, DBA can be expected as a robust alternative for pentacene.

**Table 1:** OTFTs parameters of C12-DBA-C12 and Cy5-DBA-Cy5

Compound	*μ*_max_/*μ*_avg_^a^ (cm^2^ V^−1^ s^−1^)	*I*_on_/*I*_off_^b^	*V*_th_ c (V)
C12-DBA-C12	2.97/1.02	10^6^	3–25
Cy5-DBA-Cy5	0.84/0.45	10^5^–10^6^	17–22

a Average mobility evaluated using data from 20 and 6 devices, respectively.

b On/off current ratio determined from the *I*_D_–*V*_G_ characteristics.

c Threshold voltage of devices estimated by the |*I*_D_|^1/2^*versus V*_G_ plots to *I*_D_ = 0 A.

By the way, the transfer curves of their devices exhibited unwanted hysteresis due to defect states at various interfaces (Fig. S4†), which is associated with defect states at the semiconductor–dielectric and metal–semiconductor interfaces. Further device optimization is under way in our laboratory.

### Thin-film morphologies and structural analyses

**Fig. 6:**
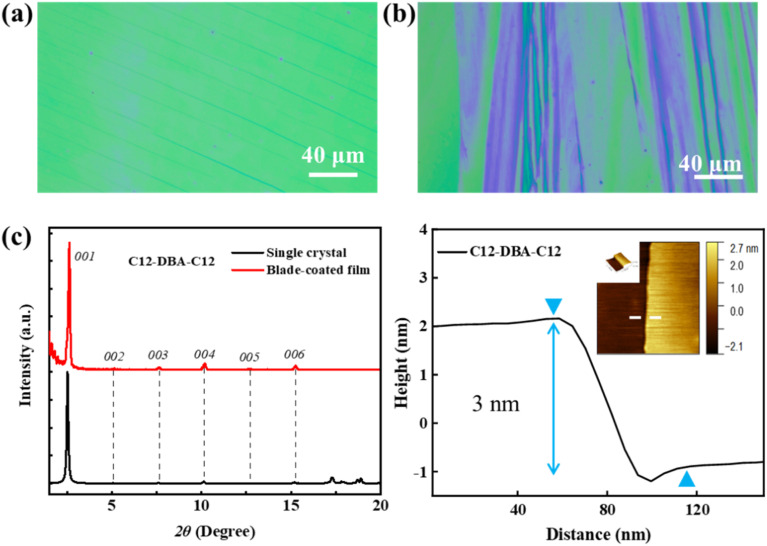
Optical microscopy images of (a) C12-DBA-C12 and (b) Cy5-DBA-Cy5, (c) XRD patterns and AFM topographic image and corresponding cross-sectional height profile of the blade-coated C12-DBA-C12 film on the BCB/SiO_2_ dielectric.

Optical microscopy, X-ray diffraction and tapping-mode atomic force microscopy (AFM) were used to examine the thin-film morphologies and molecular orientation of C12-DBA-C12 and Cy5-DBA-Cy5 in order to gain a better understanding of their carrier-transport properties. The blade-coating method enabled the formation of crystalline thin films for both compounds. However, the difference in film-forming capacity between C12-DBA-C12 and Cy5-DBA-Cy5 was easily distinguishable from the optical images. As illustrated in Fig. 6a and b, C12-DBA-C12 displayed consistent color in its large crystalline regions, whereas Cy5-DBA-Cy5 showed varied color distribution in its multiple elongated crystalline domains. Clear Bragg reflections were observed in the C12-DBA-C12 films. As exhibited in Fig. 6c, a series of intense and assignable (00*l*) reflections in the XRD patterns were well-indexed to the crystallographic *ab* cell, indicating a highly crystalline layered structure of C12-DBA-C12 thin films on the BCB/SiO_2_ substrate. The interlayer distance was estimated to be 33.56 Å, which coincided with the step height of approximately 3 nm in its AFM height profile (Fig. 6c). In contrast, in Fig. S4† only one weak diffraction peak was detected for Cy5-DBA-Cy5, suggesting that its film had low crystallinity and thus lead to relatively poor transistor performance. These results demonstrate that the enhanced electrical characteristics of C12-DBA-C12 OTFTs originate from its uniform and highly-layered crystalline film.

## Conclusion

In this study, we successfully synthesized two alkylated DBA derivatives of C12-DBA-C12 and Cy5-DBA-Cy5 with linear *n*-dodecyl and pentyl-cyclohexyl chains. They both exhibited excellent thermal stability, with *T*_d_ surpassing 395 °C. Notably, C12-DBA-C12 had a *T*_d_ of 434 °C. C12-DBA-C12 and Cy5-DBA-Cy5 provided distinct types of packing motifs, depending on their different alkyl structures. C12-DBA-C12 crystallized into the 2D herringbone packing with a lamellar structure, while Cy5-DBA-Cy5 formed the alternated packing with π-cores and alkyl chains interdigitated with each other in the molecular long axis. The observed packing difference is clearly associated with their film crystallinity and OTFT characteristics. C12-DBA-C12 readily formed high-quality uniaxially-oriented crystalline films that exhibited high mobility up to 2.97 cm^2^ V^−1^ s^−1^, while Cy5-DBA-Cy5 showed relatively poor film crystallinity and moderate mobility around ∼0.45 cm^2^ V^−1^ s^−1^. The pentyl-cyclohexyl-substituted Cy5-DBA-Cy5 didn't demonstrate the expected molecular packing and film crystallinity, which is associated with the short C–H⋯π contacts (2.882 Å) between cyclohexyls and π-cores hindering the formation of π–π stack. We believed that these findings enable DBA to be explored further as a pentacene alternative for creating high-performing OSCs in organic electronics, and can offer guidance for regulating molecular packings by alkyl engineering.

## Experimental section

### General methods

^1^H NMR and ^13^C NMR were recorded in CDCl_3_ using a Bruker Advance III HD spectrometer at 500 MHz. High-resolution mass spectra (HRMS) were recorded on Thermo EI mass spectrometer (DSQ II). The absorption spectra were detected by UV-vis-NIR spectrophotometer (PerkinElmer, Lambda 1050). Thermogravimetric analyses (TGA) were performed with PE Pyris1 TGA under a nitrogen atmosphere with a heating rate of 10 °C min^−1^ from 30 to 780 °C. The polarized optical microscopy (POM) observations were conducted on Olympus BX53. Atomic force microscopy (AFM) measurements were carried out in trapping mode on Park NX20. X-ray diffraction (XRD) was performed on Rigaku Ultima IV (Cu Kα, *λ* = 0.15443 nm). The OTFTs characteristics were measured using a semiconductor parametric analyzer (Keithley 4200A-SCS).

### Materials synthesis

Unless otherwise noted, all the reagents and solvents used in this study were purchased from Energy Chemical, Macklin Biochemical and Sigma-Aldrich Chemical. Two R-DBA-R products were prepared by the similar way, and in this case the templated synthesis of C12-DBA-C12 was introduced as an example.

#### 4,4′′-Didodecyl-[1,1':4′,1′′-terphenyl]-2′,5′-dicarbaldehyde (1a)

2,5-Dibromoterephthalaldehyde (1.46 g, 5 mmol), (4-dodecylphenyl)boronic acid (11 mmol, 3.2 g) Pd (PPh_3_)_4_ (0.58 g, 0.5 mmol), K_2_CO_3_ (4.14 g, 30 mmol), toluene (60 mL) and H_2_O (30 mL) were placed into a three necked-flask, and the mixture was heated to reflux for 10 h. After cooling, the mixture was extracted with CH_2_Cl_2_, dried by Na_2_SO_4_, and evaporated *in vacuo*. The resulting solid was collected and purified by column chromatography using hexane-CH_2_Cl_2_ to afford final product 1a (1.87 g, 60%). ^1^HNMR (500 MHz, CDCl_3_) 10.09 (s, 2H), 8.09 (s, 2H), 7.33 (m, 8H), 2.69 (m, 4H), 1.68 (m, 4H), 1.30 (m, 36H), 0.88 (t, 6H).

#### 4,4′′-Didodecyl-2′,5′-bis-2-methoxyvinyl)-1,1′:4′,1′′-terphenyl (2a)

(Methoxymethyl)triphenylphosphonium chloride (2.6 g, 7.53 mmol), *t*-BuOK (0.84 g, 7.5 mmol) and dry THF (50 mL) were placed into a three-necked flask, then compound 1a (1.17 g, 1.88 mmol) in THF (10 mL) was injected into the mixture, and the resultant solution was stirred at room temperature overnight. After the reaction terminated, the mixture was diluted with water, extracted with ethyl acetate, dried by MgSO_4_, and evaporated *in vacuo*. The resulting solid was collected and purified by column chromatography using hexane–CH_2_Cl_2_ were afforded (0.83 g, 1.24 mmol, 65.3%). Compound 2a was a mixture of multiple *E* and *Z*-isomers, and the exact chemical shifts of protons were difficult to be determined.

#### 3,10-Didodecylbenzo[*k*]tetraphene (C12-DBA-C12)

MeSO_3_H (2 mL) was added dropwise into a three-necked flask with compound 2a (1.33 g, 2 mmol) and dry CH_2_Cl_2_ (30 mL), and then the resulting mixture was stirred at 25 °C for 3 h. After reaction, CH_3_OH was poured down to acquire solid precipitates by filtration. The crude product was purified by repeated recrystallization and column chromatography to afford C12-DBA-C12 (0.86 g, 70%).

C12-DBA-C12: ^1^H NMR (500 MHz, CDCl_3_): *δ* ppm 9.10 (s, 2H), 8.77 (s, *J* = 8.4 Hz, 2H), 7.93 (d, *J* = 8.9 Hz, 4H), 7.71 (d, *J* = 9.5 Hz, 2H), 7.55 (d, *J* = 8.4 Hz, 2H), 2.84 (t, *J* = 7.7 Hz, 4H), 1.76 (dt, *J* = 15.4, 7.6 Hz, 4H), 1.57–1.10 (m, 38H), 0.87 (t, *J* = 6.9 Hz, 6H). ^13^C NMR (126 MHz, CDCl_3_) *δ* ppm 141.74, 132.09, 130.60, 128.95, 128.28, 127.79, 127.45, 127.11, 122.79, 121.84, 35.96, 31.93, 31.58, 29.69, 29.65, 29.63, 29.57, 29.41, 29.37, 22.70, 14.12. HRMS (EI): calculated for C_46_H_62_ 614.4852 found 614.487.

Cy5-DBA-Cy5: this compound was prepared by similar procedures to those of C12-DBA-C12, and the characterization data are as follows. ^1^H NMR (500 MHz, CDCl_3_): *δ* ppm 9.09 (s, 2H), 8.77 (d, *J* = 8.5 Hz, 2H), 7.93 (d, *J* = 9.0 Hz, 2H), 7.71 (d, *J* = 8.9 Hz, 4H), 7.59 (d, *J* = 8.4 Hz, 2H), 2.71 (t, *J* = 12.1 Hz, 2H), 2.71 (t, *J* = 12.1 Hz, 2H), 1.99 (dd, *J* = 49.9, 11.2 Hz, 8H), 1.62 (dt, *J* = 12.8, 9.9 Hz, 4H), 1.44–1.20 (m, 14H), 1.14 (dd, *J* = 24.5, 10.2 Hz, 4H), 0.92 (t, *J* = 7.0 Hz, 6H). HRMS (EI): calculated for C_44_H_54_ 582.4226, found 582.422.

### OTFTs fabrication

Typical bottom-gate top-contact OTFTs were fabricated as follows: heavily p-doped Si wafers with thermally-grown 300 nm SiO_2_ were used as substrates for all the devices. Firstly, the Si/SiO_2_ substrates were cleaned by ultrasonic cleaner with deionized water, acetone, and isopropanol for 10 min. Then, they were dried by vacuum oven at 100 °C for 30 min and treated by plasma cleaner for 15 min. Afterward, according to the reported literature, the surfaces of SiO_2_/Si substrates were modified with thermally crosslinked BCB polymer layer (50–60 nm). Considering the less toxic of anisole and similar film-forming ability in anisole and chlorobenzene, anisole was selected as the solvent for blade-coating crystalline films. Before fabricating the semiconductor films of C12-DBA-C12 and Cy5-DBA-Cy5, 0.05 or 0.075 wt% solutions of them in anisole were prepared at approximately 70–80 °C. Next, the blade was fixed and the stage holding the substrate moved at 2.5–5 μm s^−1^. The substrate was kept on a hot plate with temperature 70 °C and solution was injected into the gap space of about 100 μm between blade and substrate. Finally, 50 nm Au as source/drain electrodes was thermally evaporated on the semiconductor films of C12-DBA-C12 and Cy5-DBA-Cy5 through a shadow mask. The source/drain electrodes in each of these devices were constructed with channels that were parallel to the shearing direction. Field effect mobilities were calculated from the saturation transfer characteristics according to the following equation:*I*_DS_ = (*WC*_*i*_/2*L*)*μ*(*V*_G_ − *V*_th_)^2^where *C*_*i*_ is the capacitance of the dielectric layer; *I*_DS_ is the source–drain current, and *V*_D_, *V*_G_, and *V*_th_ are the source-drain, gate, and threshold voltages, respectively.

## Data availability

The data that support the findings of this study are available from the corresponding author, Tengzhou Yang (E-mail: yangtz@qfnu.edu.cn), upon reasonable request.

## Author contributions

Haoming Wei analyzed the XRD and AFM results. Bingqiang Cao designed and analyzed the research. Tengzhou Yang provided funding support, analyzed research results and wrote the manuscript.

## Conflicts of interest

There are no conflicts to declare.

## Supplementary Material

RA-014-D4RA07439D-s001

RA-014-D4RA07439D-s002
