# Supplementary material for: Dialkylated dibenzo[a,h]anthracenes for solution-processable organic thin-film transistors
Source: RSC Adv. 2024 Nov 14;14(49):36390–7. doi: 10.1039/d4ra07439d (PMC11561707; doi:10.1039/d4ra07439d)
Supplement: RA-014-D4RA07439D-s001 [file RA-014-D4RA07439D-s001.pdf]

Supplementary Information (SI) for RSC Advances.
This journal is © The Royal Society of Chemistry 2024

Supporting Information

Dialkylated Dibenzo[*a,h*]anthracenes for Solution-processable Organic Thin-Film Transistors

Tengzhou Yang [a], Liang Zhang, Yucong Bao, and Haoming Wei *[a]

[a.] School of Physics and Physical Engineering, Qufu Normal University, Qufu 273165, Shandong, China.

Table S1. List of solubility of C12-DBA-C12 and Cy5-DBA-Cy5

Solvent	Solubility (mg/ml, 60 °C)	
	C12-DBA-C12	Cy5-DBA-Cy5
Anisole	2.56	$<$0.9
Chlorobenzene	15.38	4.44

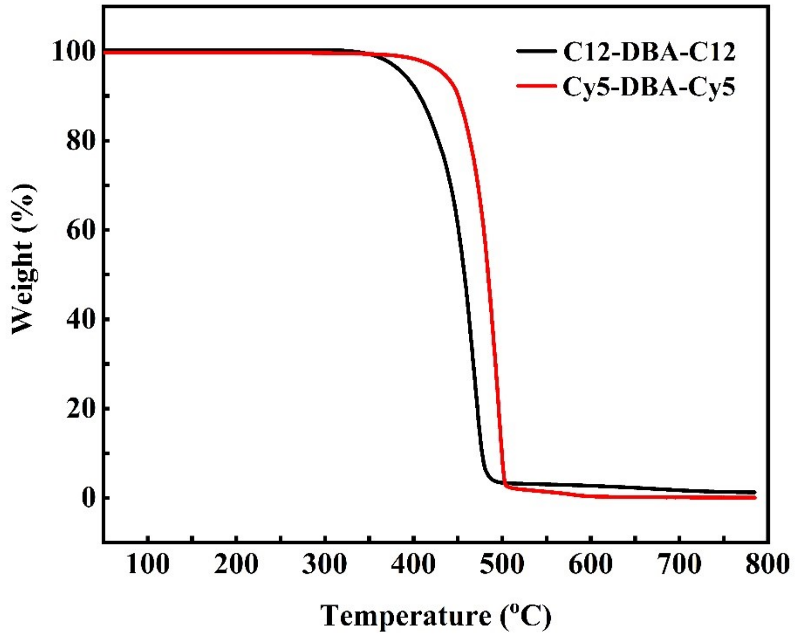

Figure S1. TGA thermograms of C12-DBA-C12 and Cy5-DBA-Cy5 at a heating rate of 10 °C/min under N_2 atmosphere

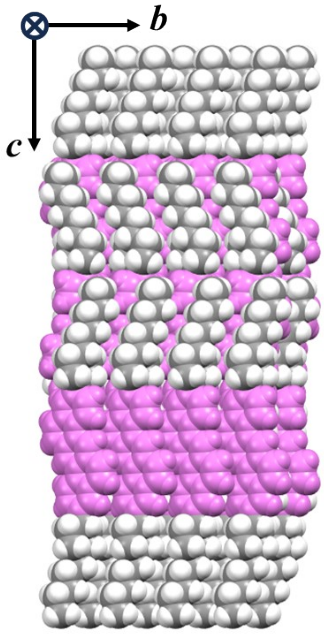

Figure S2. The molecular packing in the *b*-*c* plane of Cy5-DBA-Cy5 crystal (the DBA cores were marked in violet for clarity)

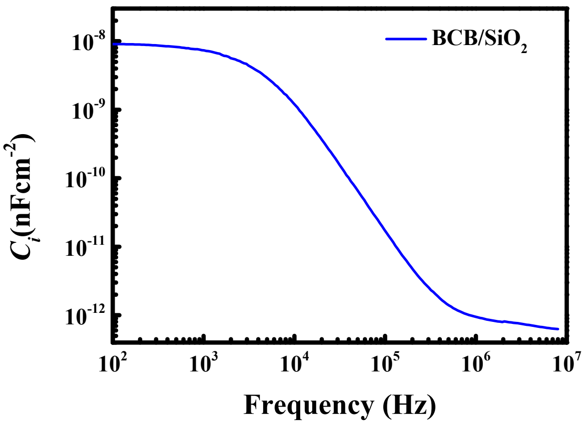

Figure S3. Frequency dependence of capacitance (C_i) for BCB/SiO_2 dielectrics measured from the sandwich structure (Au/insulator/heavily p-doped Si), showing C_i 9.1 nF cm^{-2}, at 100 Hz.

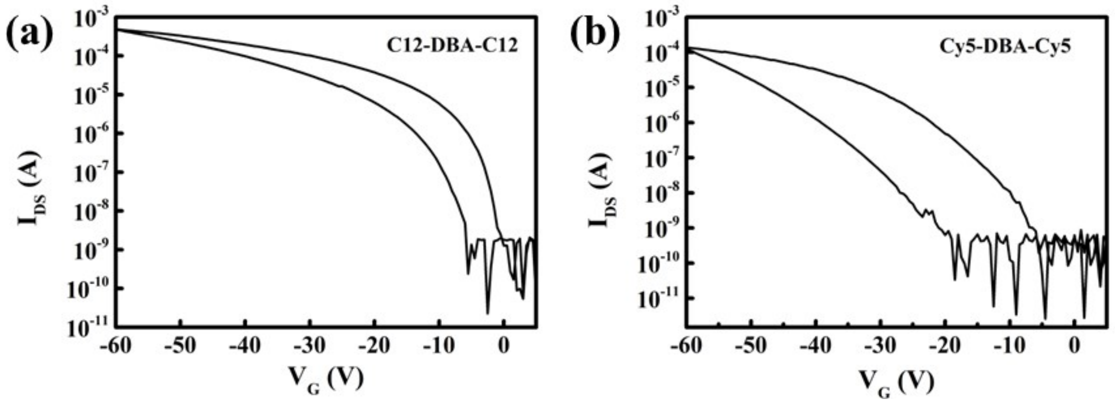

Figure S4. Transfer curves of C12-DBA-C12 and Cy5-DBA-Cy5 OTFTs during forward and the forward and reverse scanning

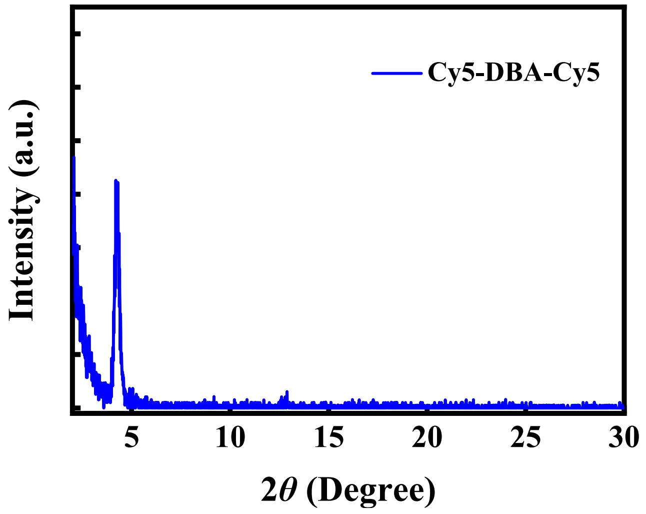

Figure S5. XRD patterns of the blade-coated Cy5-DBA-Cy5 film on the BCB/SiO_2 dielectric.

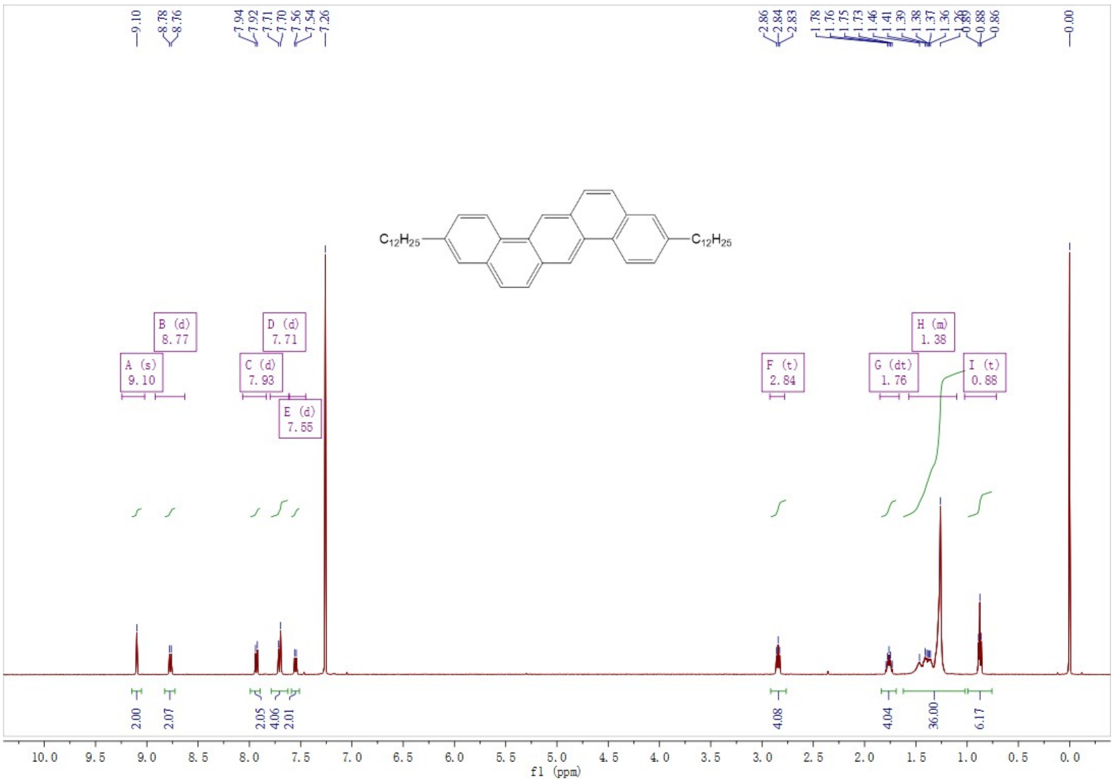

Figure S6. ^{1}H NMR spectrum of C12-DBA-Cy12

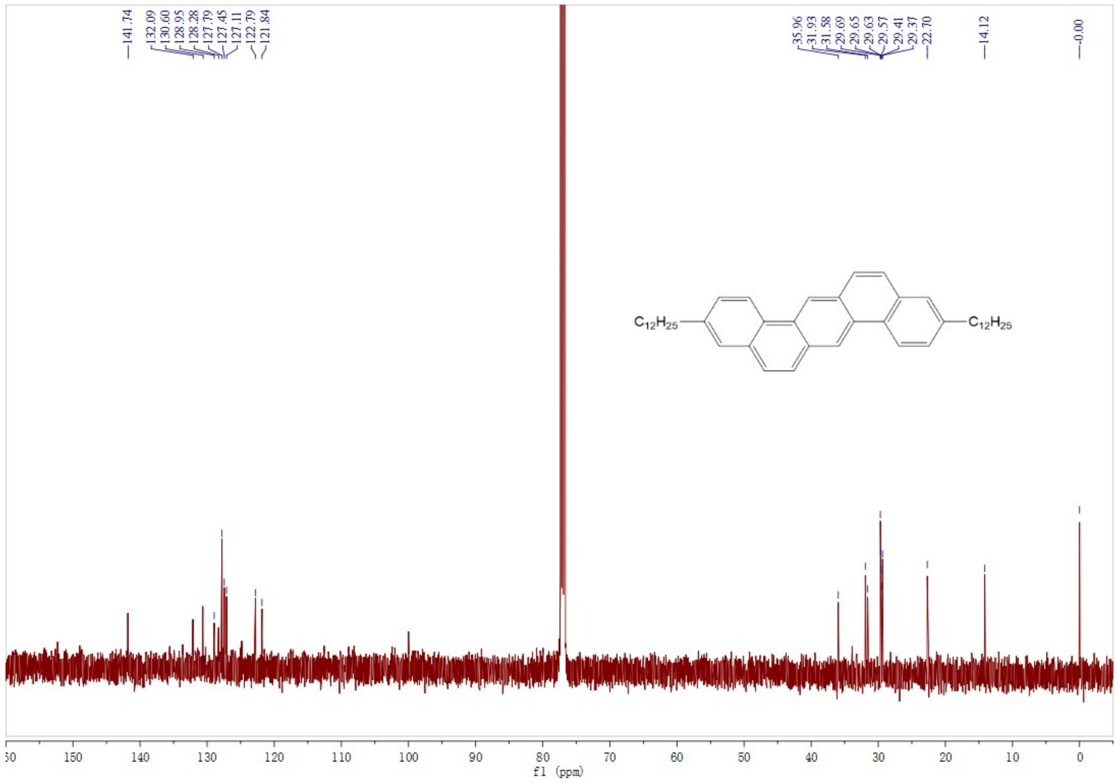

Figure S7. ^{13}C NMR spectrum of C12-DBA-Cy12

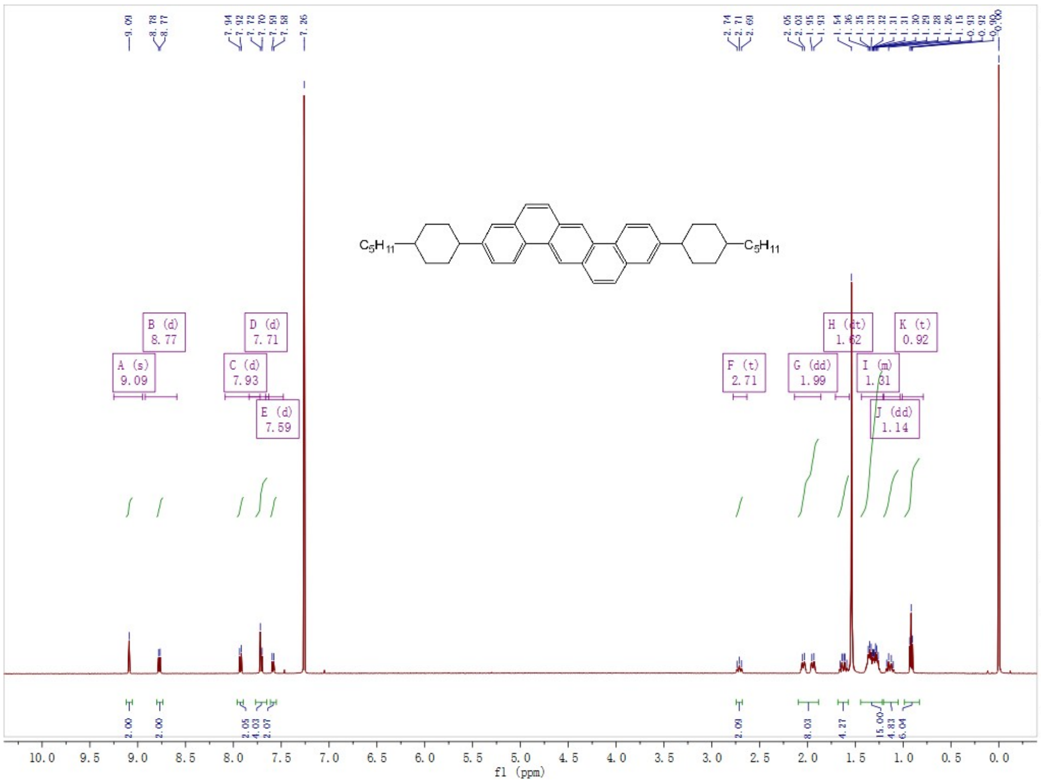

Figure S8. ^{1}H NMR spectrum of Cy5-DBA-Cy5

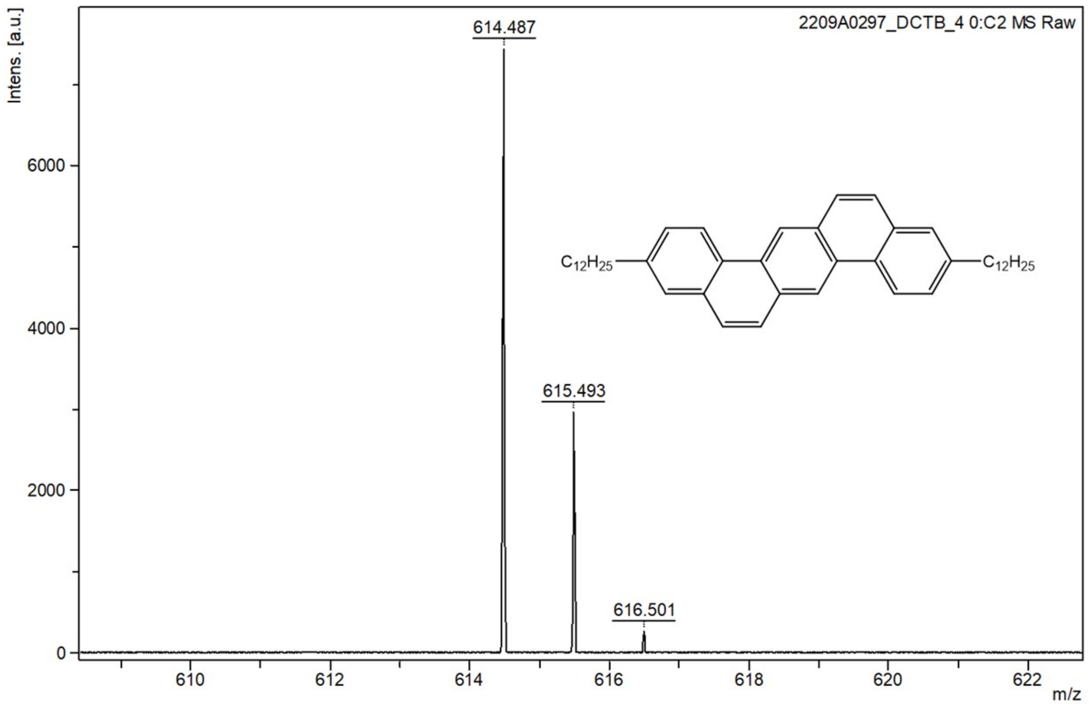

Figure S9. HRMS spectrometry of C12-DBA-C12

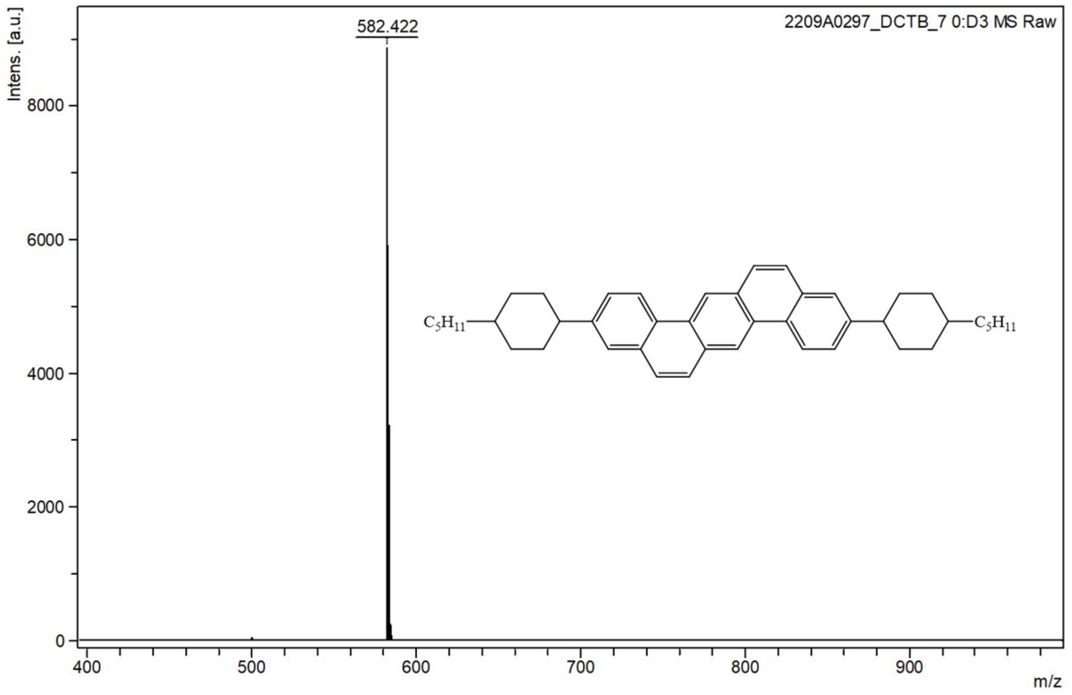

Figure S10. HRMS spectrometry of Cy5-DBA-Cy5

Table S2. Crystal structures of C12-DBA-C12 and Cy5-DBA-Cy5

**Empirical**	**C12-DBA-C12**	**Cy5-DBA-Cy5**
Formula weight	614.95	582.87
Temperature/K	295	289
Crystal system	triclinic	monoclinic
Space group	P-1	C2/c
a/Å	6.9582(3)	25.7538(5)
b/Å	7.7761(4)	5.77880(10)
c/Å	34.9263(15)	25.3638(4)
α/°	88.781(4)	90
β/°	89.088(3)	112.982(2)
γ/°	89.979(4)	90
Volume/Å^3	1889.11(15)	3475.18(12)
Z	2	4
ρ_{calc}g/cm^3	1.081	1.114
μ/mm^{-1}	0.442	0.459
F(000)	676.0	1272.0
Crystal size/mm^3	0.03 × 0.02 × 0.01	0.12 × 0.03 × 0.02
Radiation	Cu Kα (λ = 1.54184)	Cu Kα (λ = 1.54184)
2Θ range for data collection/°	5.062 to 144.232	7.572 to 155.4
Index ranges	-8 ≤ h ≤ 8, -7 ≤ k ≤ 9, -42 ≤ l ≤ 43	-31 ≤ h ≤ 32, -7 ≤ k ≤ 2, -30 ≤ l ≤ 31
Reflections collected	23926	11199
Independent reflections	7098 [R_{int} = 0.0993, R_{sigma} = 0.0605]	3527 [R_{int} = 0.0175, R_{sigma} = 0.0236]
Data/restraints/parameters	7098/0/417	3527/0/200
Goodness-of-fit on F^2	1.088	1.132
Final R indexes [I>=2σ (I)]	R_1 = 0.1174, wR_2 = 0.3056	R_1 = 0.0782, wR_2 = 0.2576

Final R indexes [all data]	R_1 = 0.1679, wR_2 = 0.3415	R_1 = 0.0863, wR_2 = 0.2708
Largest diff. peak/hole / e $Å^{-3}$	0.35/-0.29	0.60/-0.27
